# Construction and validation of steroid-induced rabbit osteonecrosis model

**DOI:** 10.1016/j.mex.2022.101713

**Published:** 2022-04-26

**Authors:** Tongtong Zhu, Mengyang Jiang, Mingran Zhang, Liguo Cui, Xiaoyu Yang, Xukai Wang, Guangyao Liu, Jianxun Ding, Xuesi Chen

**Affiliations:** a Department of Orthopedics, China-Japan Union Hospital of Jilin University, 126 Xiantai Street, Changchun 130033, PR China; b Key Laboratory of Polymer Ecomaterials, Changchun Institute of Applied Chemistry, Chinese Academy of Sciences, 5625 Renmin Street, Changchun 130022, PR China; c Department of Ophthalmology, The Second Hospital of Jilin University, 218 Ziqiang Road, Changchun 130041, PR China

**Keywords:** Osteonecrosis, Steroid, Rabbit, Bone, Animal model

## Abstract

Osteonecrosis is a common orthopedic disease in clinic, resulting in joint collapse if appropriate treatment is not given in time. The clinical usage of high-dose steroid is one of the common causes of osteonecrosis. In several studies, the intravenous injection of steroid with or without lipopolysaccharide is the most commonly used strategy to construct osteonecrosis animal model. However, the injection dose, frequency, and interval of steroid and validation of successful model construction lack generally accepted protocol, and the survival and model formation rates are unsatisfactory. We have optimized the construction protocol of osteonecrosis animal model based on the previously reported ones and established a mature animal model of osteonecrosis for future studies.

• A rabbit model of osteonecrosis was constructed by multiple injections of high-dose methylprednisolone.
• The multidisciplinary biomedical examinations demonstrated the successful construction of osteonecrosis model in the rabbit.

## Graphical abstract

**Figure:**
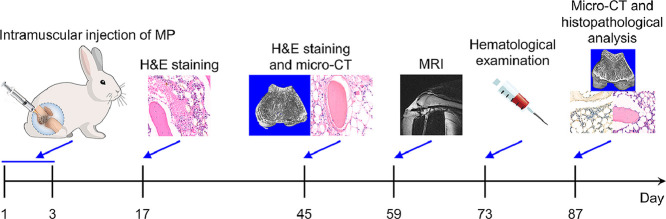

Specifications table

**Table:** 

Subject Area:	Medicine and Dentistry
More specific subject area:	*Establishment of animal models of specific diseases*
Method name:	*Steroid-induced rabbit osteonecrosis model*
Name and reference of original method:	*L. Qin, G. Zhang, H. Sheng, K.W. Yeung, H.Y. Yeung, C.W. Chan, W.H. Cheung, J. Griffith, K.H. Chiu, K.S. Leung, Multiple bioimaging modalities in evaluation of an experimental osteonecrosis induced by a combination of lipopolysaccharide and methylprednisolone, Bone 39(4) (2006) 863−871; G. Motomura, T. Yamamoto, T. Irisa, K. Miyanishi, K. Nishida, Y. Iwamoto, Dose effects of corticosteroids on the development of osteonecrosis in rabbits, J. Rheumatol. 35(12) (2008) 2395−2399.*
Resource availability:	*Department of Orthopedics, China-Japan Union Hospital of Jilin University; Key Laboratory of Polymer Ecomaterials, Changchun Institute of Applied Chemistry, Chinese Academy of Sciences*

## Background

**Scheme 1:**
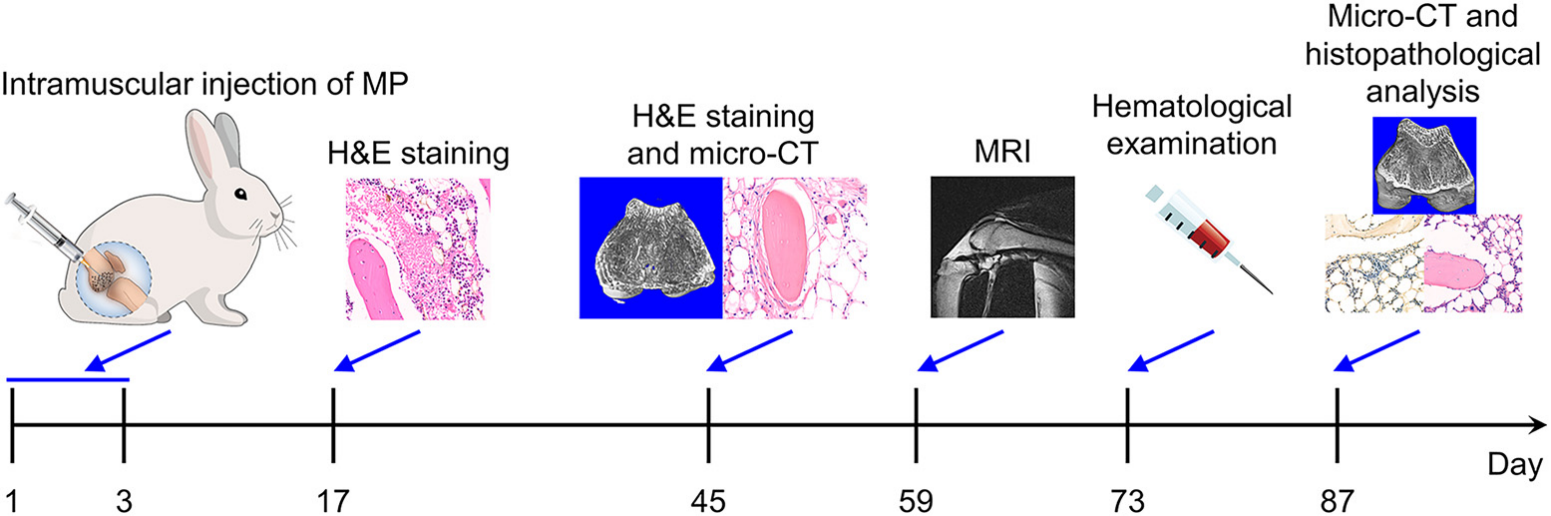
Timeline of construction and validation of steroid-induced rabbit osteonecrosis model.

Osteonecrosis is a common disease in clinical orthopedics, which may cause by many factors, including trauma, steroid therapy, alcoholism, and so forth [1]. Steroids are one of the most common and severe inducements of osteonecrosis in clinic after treating many clinical diseases, such as severe acute respiratory syndrome, systemic lupus erythematosus, and rheumatoid arthritis [2], [3], [4], [5]. Reliable animal models with excellent parallelism and repeatability are the basis of high-level biomedical research [6]. In the studies of osteonecrosis, steroid induction was commonly used to establish the osteonecrosis rabbit model, but without an authoritative and mature protocol. One of the modeling methods was to inject lipopolysaccharide (LPS) with a dose of 10.0 µL per kg body weight and then three consecutive times of methylprednisolone (MP) with a dose of 20.0 mg per kg body weight (mg (kg BW)^−1^) and each time interval of 24 h. The other one was to inject MP once with a dose of 20.0 mg (kg BW)^−1^ [7,8]. After trying and analyzing the two most used experimental methods, a more reliable modeling method of three injections of a high-dose methylprednisolone was developed to construct an osteonecrosis animal model with improved survival rate and model formation rate (Scheme 1), which will promote the preclinical and clinical research of osteonecrosis treatment [9].

## Method details

### Materials

MP was purchased from Pfizer (Belgium). Xylazine hydrochloride was purchased from Shengda Animal Medicine Co., Ltd. (Dunhua, P. R. China). Paraformaldehyde (PFA) and ethylenediaminetetraacetic acid (EDTA) were provided by Aladdin Biochemical Technology Co., Ltd. (PFA, C104190, EDTA, E118595, Shanghai, P. R. China). The enzyme-linked immunosorbent assay (ELISA) kits were bought from Anoric Biotechnology Institute (osteocalcin (OCN), TAE-458R, bone-specific alkaline phosphatase (B-ALP), TAE-168R; Tianjin, P. R. China). The sources of primary antibodies were listed as follows: αOCN (1:100, ab13418, Abcam, Cambridge, UK) and αRunx2 (1:200, bs-1134R, Bioss, Beijing, P. R. China).

### Construction of a rabbit osteonecrosis model

All animal experiments were performed in compliance with the principles of the Institutional Animal Care and Use Committee of Jilin University. A total of 21 adult male Japanese white rabbits (aged 22−26 weeks old and weighing 2.8−4.0 kg) were fed with a standard laboratory diet at the Animal Center of Jilin University.

The rabbit osteonecrosis model was constructed according to previously reported protocols with appropriate modifications [10,11]. In detail, the model was established by intramuscular injection of 40.0 mg (kg BW)^−1^ MP into the right gluteus medius every day for three times. At each pre-determined time point (2, 6, 8, 10, or 12 weeks), the hematological test, radiologic analysis, and histopathological examination were performed to monitor the pathogenesis of osteonecrosis.

### Hematological examination of osteonecrosis model

OCN and B-ALP, two serum markers of bone formation, were detected using ELISA kits at 10 weeks after induction of osteonecrosis. Blood samples were collected from the auricular arteries after the animals fasted for 12 h. The whole blood samples were clotted in a water bath for about 30 min at 37 °C and then overnight at 4 °C. After centrifugation for 5 min at 1500 r min^−1^, blood serum was isolated and preserved at −80 °C. All procedures were strictly performed according to the protocols provided by the manufacturers. Using a microplate reader, we measured the absorbance value at 450 nm of the samples (SPARK, TECAN, Switzerland). All samples were assayed in triplicate.

### Magnetic resonance imaging and micro-computed tomography analysis

Eight weeks after induction of osteonecrosis, six rabbits were randomly selected and anesthetized with 10.0 mg (kg BW)^−1^ of xylazine hydrochloride intramuscularly. All the femoral condyles of rabbits were imaged on a Siemens Skyra 3.0T MR scanner (Berlin, Germany). The parameters were listed as follows: T1-weighted image, TR 634 ms, TE 47 ms, T2-weighted image, TR 3000 ms, TE 71 ms, FoV read 70 mm, FoV phase 100%, slice thickness 2.0 mm, Voxel size 0.2 × 0.2 × 2.0 mm^3^, and base resolution 320.

Two weeks after induction of osteonecrosis, three rabbits in the model group were sacrificed. Six weeks after induction of osteonecrosis, three rabbits in the normal group and three rabbits in the model group were sacrificed. Twelve weeks after induction of osteonecrosis, three in the normal group and six rabbits in the model group were sacrificed. After being fixed with 4% (*W*/*V*) PBS-buffered PFA, femoral condyles of all the rabbits were processed for micro-CT and histopathological analysis. Changes in the cancellous bones of the femoral condyles were assessed *via* radiographic examination using micro-CT (Skyscan1172, Bruker, Karlsruhe, Germany). The specimens were scanned at 9 µm camera pixel size, 27.22 µm pixel size, 80 kV, and 100 µA. Data sets were acquired at 0.6° steps over a 180° angular range. The numbers of rows and columns were 666 and 1000, respectively. After scanning, the data were reconstructed by NRecon software with 192.60° reconstruction angle, and the three-dimensional (3D) reconstruction of the femoral condyle was processed by CT-vox (Version: 3.1.1, Skyscan1172, Bruker, Karlsruhe, Germany). The area of interest (ROI) was set as the cancellous bone area (5.2 mm in diameter) in the femoral condyle, and the bone morphological parameters in the ROI were calculated using CT-An software (Version: 1.15.4.0, Skyscan1172, Bruker, Karlsruhe, Germany). Percent bone volume (BV/TV, %), trabecular number (Tb.N, mm^−1^), trabecular thickness (Tb.Th, mm), and trabecular separation (Tb.Sp, mm) were measured.

### Histopathological and histometric analysis

Ethylenediaminetetraacetic acid (EDTA, 10%, *W*/*V*) solution was used to decalcify the harvested bones, which were then embedded in paraffin blocks and cut into 5 µm thick sections for hematoxylin and eosin (H&E) staining at each pre-determined time point (2, 6, or 12 weeks) and immunohistochemistry staining at 12 weeks. The expression of OCN and runt-related transcription factor 2 (Runx2) within implantation regions were detected with the primary antibody.

### Statistical analysis

Data were presented as the mean ± standard deviation (SD). The analysis was performed using one-way ANOVA and Student's *t*-tests (Version 6.01 software, GraphPad Software, Inc., USA). *P < 0.05 was considered to be statistically significant, and **P < 0.01, ***P < 0.001, and ****P < 0.0001 were considered to be highly significant.

## Method validation

Two rabbits in the model group died on 3 days and 10 weeks after induction. One rabbit developed paralysis of the lower extremities, and they were excluded from the model group. The survival rate of animals was 86.67%. Compared with the LPS+MP method, the survival rate was significantly increased. After 2, 6, 8, 10, and 12 weeks post-injection, the successful construction of rabbit osteonecrosis model was confirmed by histopathological, imaging, and hematological analysis.

### Magnetic resonance imaging and external image analysis

**Fig. 1:**
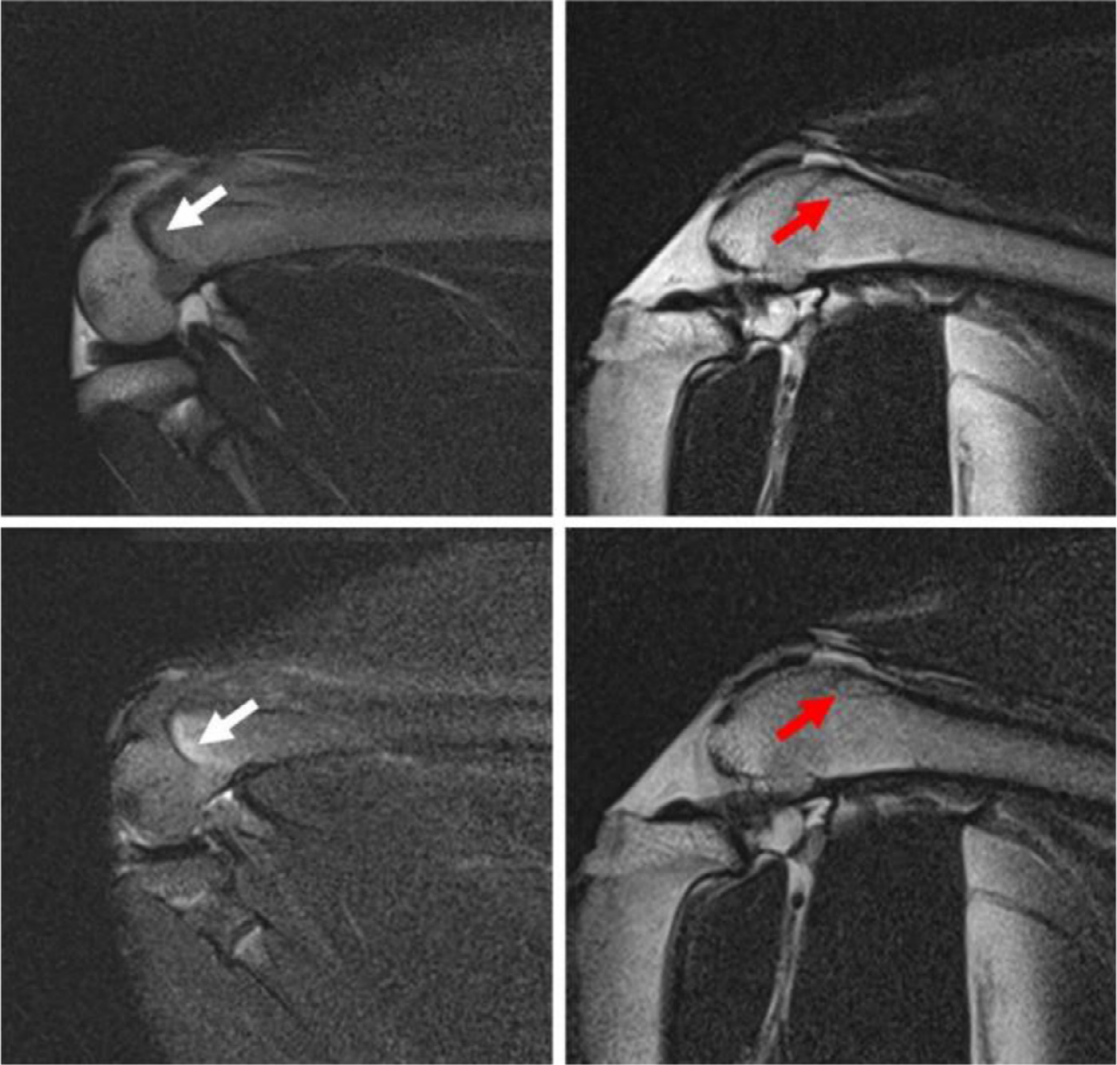
MRI images of rabbit osteonecrosis model after eight weeks. The white arrow indicates the lipedema signal, and the red arrow indicates the necrotic area.

**Fig. 2:**
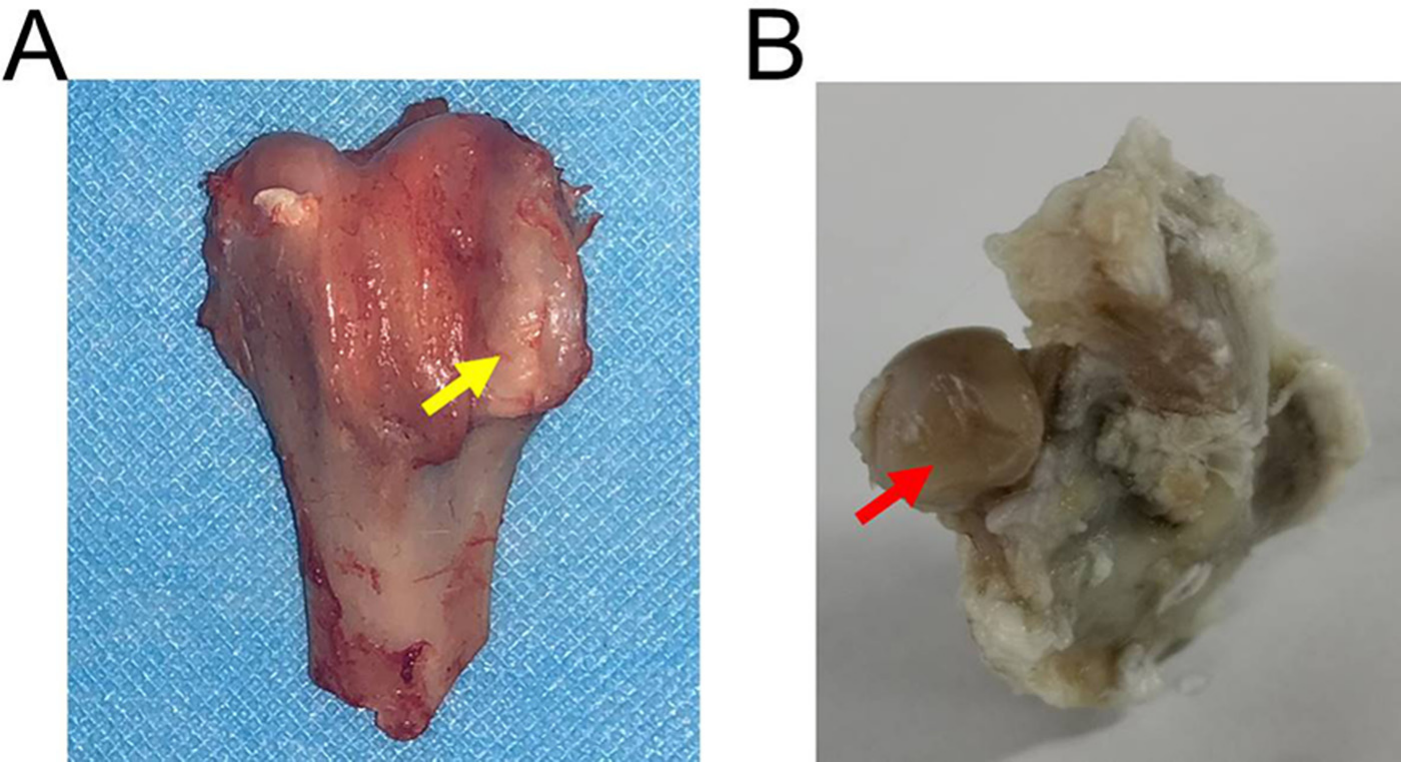
Appearance photos of femoral condyle and femoral head. (A) Change of femoral condyle. The yellow arrow indicates arthritis manifestation. (B) Change of femoral head. The red arrow indicates articular surface collapse.

The femoral condyles showed abnormal magnetic resonance imaging (MRI) signals, manifested as lipedema and linear low signal areas, which indicated the formation of osteonecrosis (Fig. 1). Additionally, the rabbit's articular surface collapsed and showed manifestations of arthritis in the osteonecrosis model (Fig. 2). Articular surface collapse and arthritis are the main secondary lesions associated with late-stage osteonecrosis, which indicates that if it is not treated in time, osteonecrosis will have severe complications.

### Micro-CT analysis

**Fig. 3:**
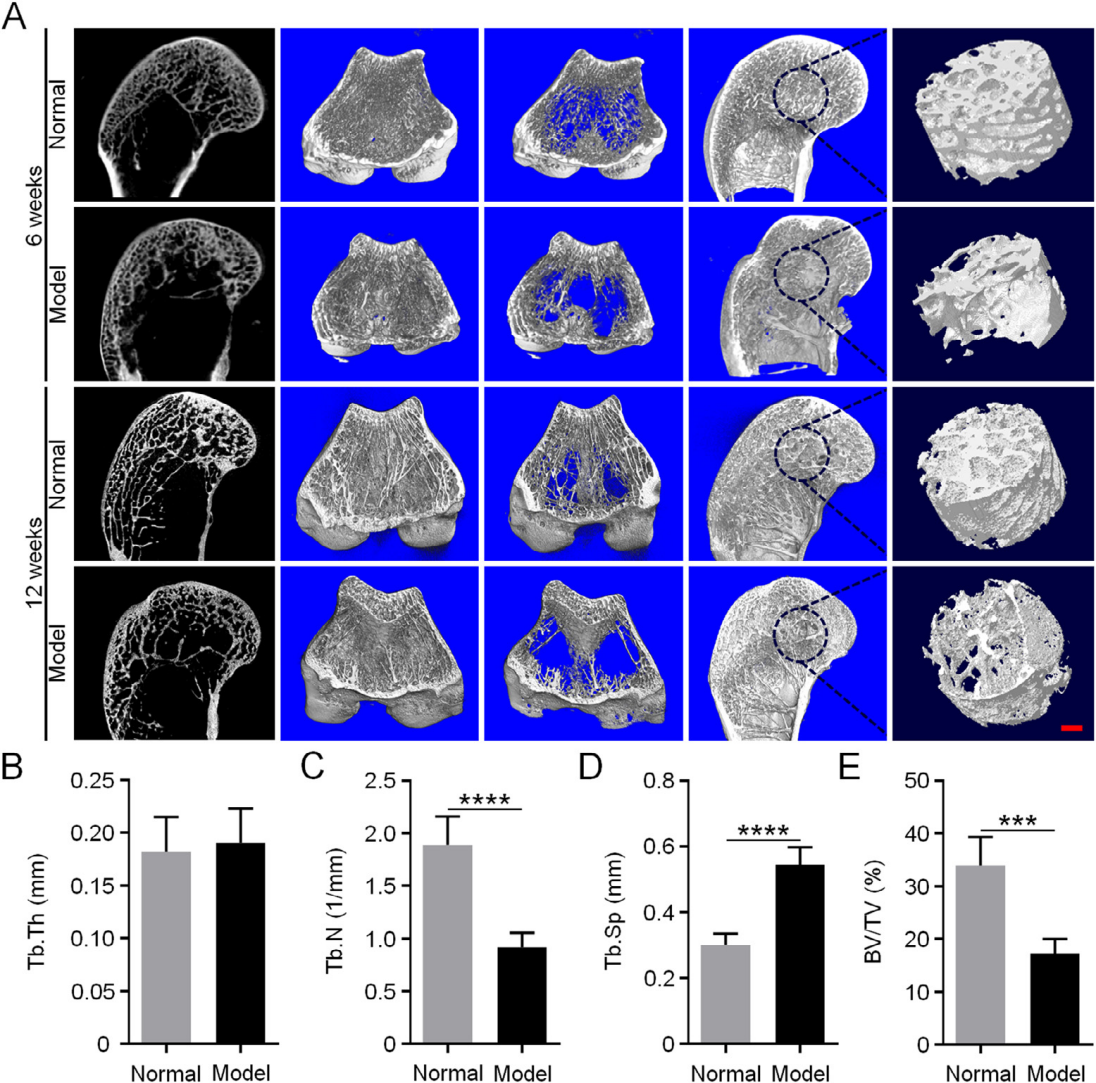
Micro-CT detection of rabbit osteonecrosis model. (A) 6 and 12 weeks after induction of osteonecrosis, routine scanning, and 3D reconstruction images of micro-CT in rabbit femoral condyles. Scale bar = 2 mm. The red circle represents ROI (*d* = 5 mm). (B–E) Quantitative analysis of micro-CT of bone microstructural parameters in ROI, Tb.Th (B), Tb.N (C), Tb.Sp (D), and BV/TV (E). Data are represented as mean ± SD (*n* = 5; ***P < 0.001, ****P < 0.0001).

Osteonecrosis always affects the activity and differentiation of osteoblasts and impacts the function of osteoclasts, leading to a destruction of the balance between the two main cell types [12,13]. The differentiation behavior of BMSCs is also disturbed by osteonecrosis, showing downregulated osteogenic differentiation and upregulated fat differentiation [14]. These factors eventually lead to decreased bone mass and thinning of bone trabeculae or even collapse [15]. The micro-CT images of the subchondral cancellous bone showed that the model group's bone mass decreased, the trabecular bone became thinner, and the structure was looser than the normal group (Fig. 3A). For the microstructural parameter Tb.Th, there was no significant difference between the normal and model groups. However, Tb.N (*P* < 0.001) and BV/TV (*P* < 0.005) of the model group were significantly decreased to approximately half compared with those of the normal group. While Tb.Sp (*P* < 0.001) of the model group was significantly increased by two times compared with the normal one (Fig. 3B–D).

### Hematological and immunohistochemical analysis

**Fig. 4:**
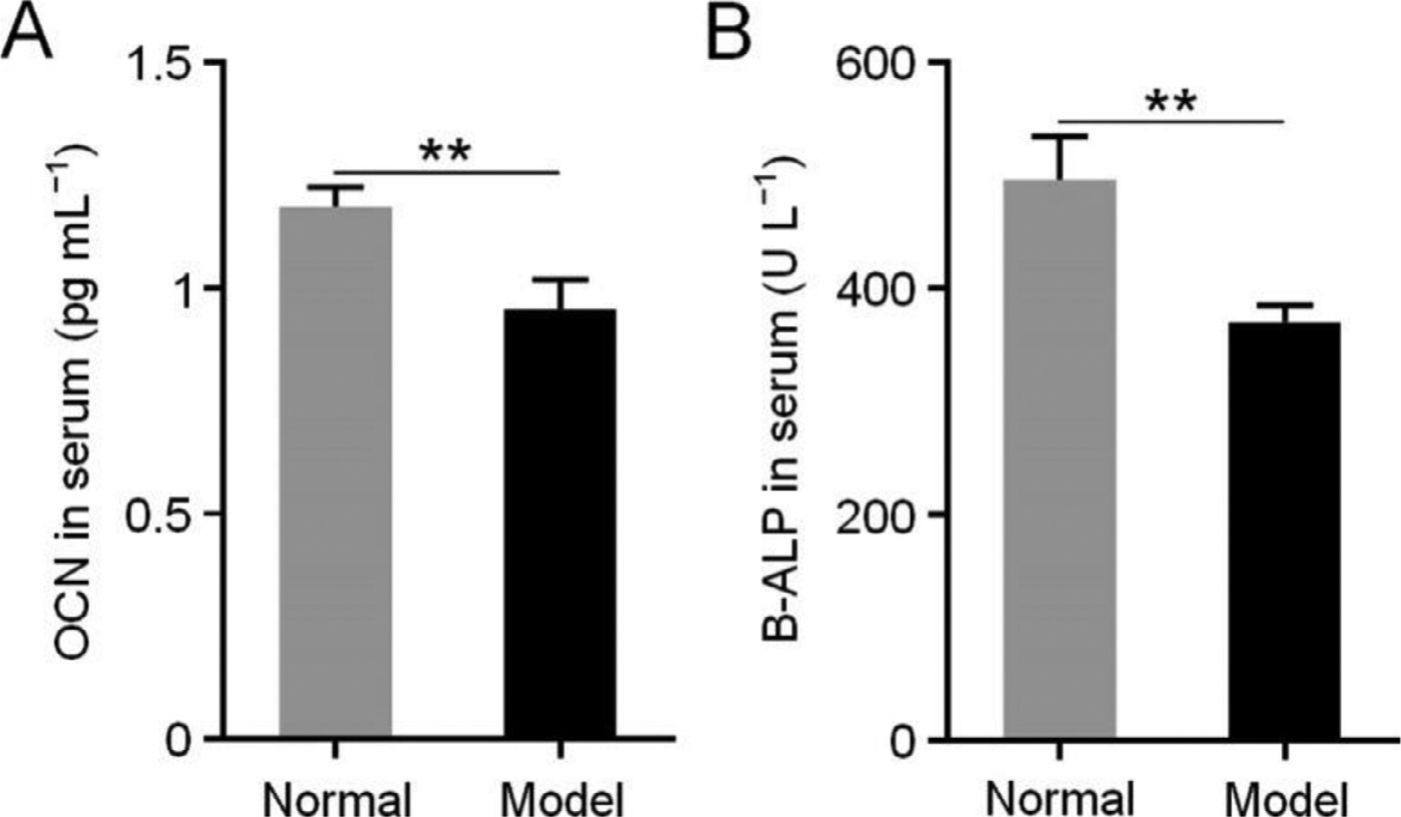
Concentrations of OCN (A) and B-ALP (B) in serum after induction of osteonecrosis. Data are represented as mean ± SD (*n* = 3; **P < 0.01).

**Fig. 5:**
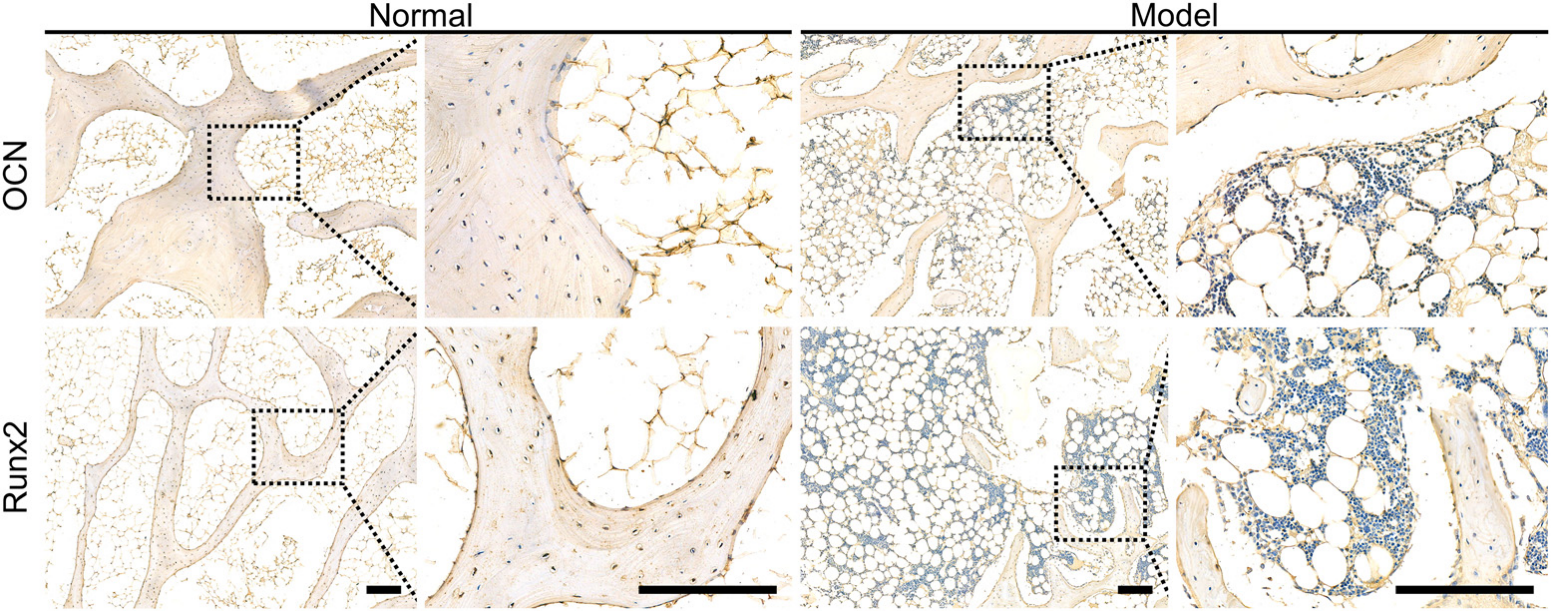
OCN and Runx2 expression levels detected by immunohistochemical analysis after induction of osteonecrosis. Scale bar = 200 µm. The black square indicates an enlarged area.

Moreover, the levels of B-ALP (Fig. 4A) and OCN (Fig. 4B) in the rabbit serum of model group decreased significantly compared with those of the normal group (*P* < 0.01) [16,17]. Furthermore, excessive steroid therapy resulted in a down-regulation of the expression of many proteins beneficial for bone regeneration, such as OCN and Runx2 [18,19]. The immunohistochemical results confirmed this finding at 12 weeks post-modeling (Fig. 5).

### Histopathological analysis

**Fig. 6:**
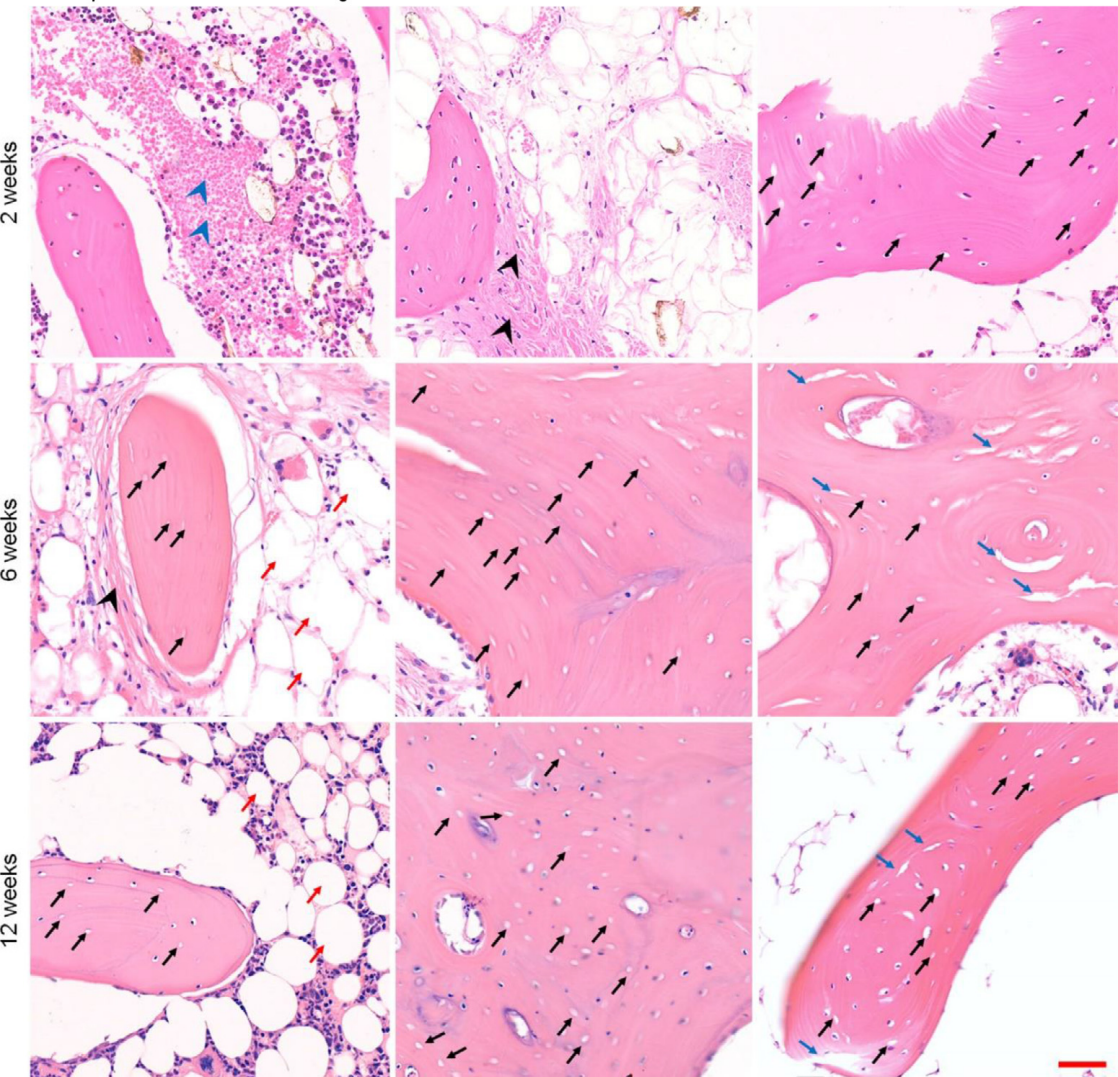
Histopathological analysis after induction of osteonecrosis. Scale bar = 50 µm. The blue arrowhead indicates bone marrow necrosis. The black arrow indicates empty lacunae. The black arrowhead indicates fiber granulation tissue. The red arrow indicates fat cells. The blue arrow indicates trabecular fracture.

Histopathological examination is the gold standard for the diagnosis of osteonecrosis. We sacrificed rabbits at 2, 6, and 12 weeks, removed the bilateral femoral condyles and femoral heads, and performed H&E staining of the samples after fixation, decalcification, and embedding. The pathologists diagnosed osteonecrosis, and H&E staining images demonstrated many typical empty lacunae in the lamellar bone trabeculae, and bone marrow necrosis appeared around. Additionally, fat cells in the surrounding bone marrow seemed to be enlarged, and bone marrow necrosis appeared. Osteoblasts appeared around the necrotic trabecular bone, suggesting that limited repair occurred. Simultaneously, the accumulation of fibrous tissue was observed, indicating the appearance of destructive repair, which was in line with the early histopathological changes of osteonecrosis. Six and twelve weeks after the induction of osteonecrosis, large numbers of typical empty lacunae could still be observed. The increase in fat cells in the bone marrow was more evident than before, and the available space for bone marrow components was significantly reduced (Fig. 6). The results indicated that the necrotic area could not be self-healed.

According to the results of histopathological examination, all rabbits with at least one osteonecrosis lesion in the examination were defined as positive, while those with no osteonecrosis lesions were negative. Our model-building success rate was 83.33%, and the mortality of animals was low.

## Discussion

The above results demonstrated that a rabbit osteonecrosis animal model was successfully established by continuous injection of large doses of MP. This method has the advantages of a high molding rate, low animal mortality, and simple and quick operation. This animal model was helpful for further basic experimental research on osteonecrosis and contribute to the advancement of osteonecrosis therapy in clinic.

## Direct submission or co-submission

Co-submissions are papers that have been submitted alongside an original research paper accepted for publication by another Elsevier journal.
